# Rejoinder: The Future of Reliability

**DOI:** 10.1007/s11336-021-09807-9

**Published:** 2021-09-17

**Authors:** Klaas Sijtsma, Julius M. Pfadt

**Affiliations:** 1 Department of Methodology and Statistics TSB, Tilburg University, PO Box 90153, 5000LE Tilburg, The Netherlands; 2 Ulm University, Ulm, Germany

**Keywords:** coefficient $\alpha$, measurement precision, reliability, statistical power

## Abstract

In this rejoinder, we examine some of the issues Peter Bentler, Eunseong Cho, and Jules Ellis raise. We suggest a methodological solid way to construct a test indicating that the importance of the particular reliability method used is minor, and we discuss future topics in reliability research.

## Introduction

Sijtsma’s discussion of coefficient $\alpha$ (Sijtsma, 2009) clarified that contrary to common belief coefficient $\alpha$ is not an index of the factor composition of an item set but rather a lower bound to a test score’s reliability and that greater lower bounds exist. Sijtsma and Pfadt’s sequel article (Sijtsma & Pfadt, 2021) discussed popular claims that reliability lower-bound methods such as coefficient $\alpha$ are not useful as test score quality measures and that theoretically coefficient $\alpha$ may even exceed test score reliability. We argued both claims are incorrect; lower bounds are useful and coefficient $\alpha$ cannot exceed reliability. We thank Peter Bentler, Eunseong Cho, and Jules Ellis for their valuable and instructive comments on the latter discussion paper. We noticed that the discussants bring up other important issues related to coefficient $\alpha$, reliability, and underlying sampling models. In our rejoinder, we first examine some of the issues they raise. Second, we explain a methodological solid way to construct a test that suggests that the particular reliability method used is of minor importance. Finally, we discuss future topics in reliability research.

## Discussants’ Issues

### A Wealth of Reliability Methods and Authors

The large array of methods for determining reliability shows psychometrics’ just fascination with the topic, but also its complexity leading to a large body of literature including many useful methods and several misunderstandings. Cho (2021) is concerned with attributing psychometric findings to the author who is the actual inventor, a quest we can only support but expect is an uphill battle in the face of Stigler’s law of eponymy that no scientific discovery is named after its original discoverer (Stigler, 1980). We also support Cho’s suggestion to speak of classical test theory (CTT) reliability and factor analysis (FA) reliability and distinguish different methods as attempts to estimate these two quantities. Sijtsma and Van der Ark (2021, chap. 2) followed this approach for CTT methods except for the naming of methods where they stuck to the names as commonly used. Following Ellis, we add that one should not forget generalizability as a third reliability approach (Sijtsma & Van der Ark, 2015). Cho’s observation that coefficient $\alpha$ often has a smaller value in real data than several other methods illustrates nicely the “limited usefulness” characterization in the titles of the Sijtsma (2009) and Sijtsma and Pfadt (2021) articles: When you use coefficient $\alpha$, notice that greater lower bounds exist.

### Sample-Based Lower Bounds

Bentler (2021) correctly points out that the lower bound result for coefficient $\alpha$ is theoretical and that sample values denoted ${\hat{\alpha }}$ may overestimate reliability $\rho _{XX^{'}}$, also noted by Cho (2021) and Sijtsma and Pfadt (2021) and true for all reliability methods. Overestimation is less of a concern as sample size is large, but for small and realistic samples, the effect may be considerable. Woodward and Bentler (1978) took the hypothesized sampling distribution of ratio $\left( \frac{1-\alpha }{1-{\hat{\alpha }}} \right) \sim F_{n,m}$ with $n=(N-1)$ (*N* is sample size) and $m=(N-1)(J-1)$ (*J* is number of items) as a point of departure (Feldt, 1965) and defined probability *a* such that $P\left[ F_{n,m}\le F_{a} \right] =1-a$, $F_{a}$ is in the distribution’s upper tail, probability *a* is user defined. Then, they derived lower bound estimate $\hat{\rho }_{(1-a)}=1-(1-{\hat{\alpha }})F_{a}$ for which $P[{\hat{\rho }}_{(1-a)}\le \rho _{XX^{'}}]\ge (1-a)$. The idea is that as sample size is smaller, ${\hat{\alpha }}$ will exceed $\rho _{XX^{'}}$ more often, and ${\hat{\alpha }}$ needs a greater downward correction to control for this overestimation. This is what sample lower bound ${\hat{\rho }}_{(1-a)}$ accomplishes with probability at least $(1-a)$. All of this depends on the correctness of the sampling distribution for a particular application. Bentler (2021) advises using greater lower bounds than coefficient $\alpha$ with smaller sampling variance and refers to Li and Bentler (2011) for additional work.

Bentler also discusses work on Guttman’s $\lambda _{4}$ (Hunt & Bentler, 2015), which is the largest value of coefficient $\alpha$ for a division of the set of *J* items in two, possibly unequal-sized item subsets without overlap. Oosterwijk, Van der Ark, and Sijtsma (2017) noticed that in many data sets estimator ${\hat{\lambda }}_{4}$ excessively overestimates reliability $\rho _{XX^{'}}$. Hunt and Bentler (2015) suggested an algorithm that avoids having to consider all possible splits of the item set, for larger item sets a virtually impossible task, and that approximates the sampling distribution of ${\hat{\lambda }}_{4}$ reasonably well. Based on simulated data, they concluded that the value corresponding to the distribution’s fifth quantile, denoted $\lambda _{4(0.05)}$, guarantees quite well that one does not overestimate $\rho _{XX^{'}}$ while being quite close to $\rho _{XX^{'}}$. Interestingly, for the 1-factor case, coefficient $\alpha$ was approximately equal to $\rho _{XX^{'}}$ with acceptable sampling error. For the 2-factor case, $\alpha$ was smaller than $\rho _{XX^{'}}$ by .03 to .04 with a somewhat larger sampling error. In general, coefficient $\alpha$ was statistically outperformed by $\lambda _{4(0.05)}$. We skip results for the greatest lower bound (GLB). Cho (2021) is of the opinion that estimators must always be unbiased, in this context ${\mathcal {E}}( {\hat{\alpha }} )=\rho _{XX^{'}}$, but we notice that in real data, CTT reliability methods are lower bounds and that we have to live with that. We reiterate lower bounds provide protection against researcher optimism possibly fueled by a desire for high reliability values; also, see Bentler (2021).

### Coefficient ${\alpha }$ Cannot Be a Reliability Point Estimate Without Additional Assumptions

Suppes and Zanotti (1981) showed that not all sets of assumptions defining a model are restrictive enough to imply testable consequences in the data that can refute the model. For example, assuming local independence for a set of items does not restrict the data, and it is well known that in the context of latent class analysis one needs to restrict the number of classes to have a model for which the data can provide support or not. Similarly, in addition to assuming local independence, item response theory (IRT) models need to make assumptions about the item response functions to be testable in the data. Following Suppes and Zanotti (1981), Ellis (2021) argues that in the context of CTT, for *J* item scores, a latent variable true-score definition exists such that the assumption of uncorrelated errors of different items is always true. The assumption of uncorrelated errors therefore is an untestable part of CTT. From this result, Ellis concludes that the lower bound theorem for coefficient $\alpha$ is true and that CTT is not restrictive enough to exclude even that for a particular test reliability, $\rho _{XX^{'}}=1$. For example, knowing that $\alpha =.9$ means that reliability $\rho _{XX^{'}}$ is somewhere between this value and 1, but without further restrictions on the model one cannot know where it is in the interval. Adding the assumption of essential $\tau$-equivalence pinpoints coefficient $\alpha$ to the reliability, implying that in the population, we now have ${\alpha =\rho }_{XX^{'}}=.9$. A more restrictive latent variable model such as a factor model adds assumptions allowing in principle to test whether the model fits to real data and pinpoint a reliability result for the reliability defined for the factor model. Nevertheless, lower bound results for CTT are correct and useful, because an $\hat{\alpha }$ value of, say, .9, obtained from a reasonably large sample guarantees that $.9<\rho _{XX^{'}}$, a result satisfactory to most researchers even when they do not know the exact value.

### Domain Sampling Approach to Reliability

Ellis (2021) argues that the true score defined as the expectation of a hypothetical within-subject distribution is ill defined as long as one does not have access to repeated measures of the same person. We agree, but also notice that the within-subject model is a thought model representing the idea that measurement values such as test scores are liable to random error, a model that with human beings unfortunately is inaccessible to experimental scrutiny. We note this is also true for alternative models, such as the random sampling model (Holland, 1990), which assigns each individual one fixed observed score rather than a distribution of observed scores. Repeated administration may indeed reveal the same score, but how will one exclude memory or practice effects? Ellis further notices that true scores vary when test administration procedures vary. This is true; hence, we emphasize the importance of a consistent test administration regime also known as standardization. This regime is necessary to optimize reliability defined as the correlation between two parallel tests, usually unavailable but approximated using lower bound methods.

Generalizability theory (Brennan, 2001; Cronbach et al., 1972) offers a different thought model in which design facets such as tests can be random samples of *J* items from a hypothesized item domain. Ellis notices that for such a random item sample, coefficient $\alpha$ estimates the coefficient of generalizability providing an almost unbiased estimate of the degree to which the *J*-item test score is representative of the test score based on the full item domain. This entails a true score defined as the score on the complete item domain. Both within-subject true score and domain-sampling true score are impossible to check with real data—according to Ellis to different degrees but from our perspective both located at or close to the “unrealism” anchor of the imaginary unrealism–realism scale. In any case, both true scores are useful as thought models. In both cases, we end up with the conclusion that the simple test score is a useful measurement value (e.g., Hemker et al., 1997).

### Reliability is Population Dependent

Bentler (2021) notices that reliability and methods for estimating it vary across different populations. He discusses reliability freed of unwanted effects of gender, age, SES, education, and so on, also known as covariate-free reliability (Bentler, 2016). His approach reminds us dimly of generalizability theory in which reliability known as generalizability is estimated corrected for various sources of variance believed to influence test performance, such as item format (e.g., forced choice, constructed response, essay), administration mode (e.g., physical presence including proctoring, Internet using distance proctoring software), and test mode (e.g., paper and pencil, computer based). Population might be a factor in a typical generalizability study. These design factors explain systematic variance in a group’s test scores, and the generalizability coefficient corrects for them. Standardization reduces random error influences on an individual’s test performance, but cannot account for covariate influences.

In his comments, Bentler mentions substantively justified models representing an empirical covariance structure and later a content-motivated covariance structure or a structural equation model. He also notices that these remarks concern a topic different from the topics we discussed in our contribution, and he is right. However, leaving this topic alone would mean missing an opportunity for calling attention to the overwhelming importance of the development of substantive theory for test and questionnaire construction and its consequences for reliability estimation.

## Psychological Theory as Basis of Measurement

If you ask a psychometrician for a solution to a problem, chances are that she will come up with an equation. Similar observations can be made of members of nearly any professional group each providing solutions reflecting their specific expertise. This group specificity provides an explanation why psychologists and psychometricians—of course, exceptions noted—do not work together more closely in solving particular measurement problems. We discuss how one should develop a measurement instrument based on both areas of expertise, and notice two lines of future research connected with reliability.

The basis of any test or questionnaire used for the measurement of a psychological attribute should be a theory of that attribute. The degree to which such a theory is developed and put to the test of empirical research challenging its correctness varies across attributes. The higher the degree of development is, the more compelling and successful the operationalization of the theory into a set of measurement prescriptions including items that uniquely elicit valid responses from tested persons. For well-developed theories, developing a preliminary test or questionnaire should be obvious. The test constructor should administer such an instrument to a preferably large sample representative of the population of interest. The test constructor next fits a convenient psychometric model to the collected data, and because the underlying theory and operationalization are sound, the model should fit the data by approximation. For instruments based on a shakier basis, the psychometric analysis is less obvious, and an exploratory approach is unavoidable. Thus, psychometrics is more important as the theoretical foundation of the measurement enterprise is more uncertain, and one needs data exploration to find out about the next steps. Irrespective of the theoretical foundation, in all cases one feeds back the psychometric results to the theory and its operationalization in an attempt to improve measurement in several consecutive steps. The finding that a common factor or another one-dimensional representation describes the data well does not imply that the items measure the intended attribute unless predicted by theory. Validity research should always complement the fitting of models to data to find evidence for the hypothesis that the instrument indeed measures the attribute. Validity research does not coincide with fitting a measurement model (Lissitz, 2009; Pearl & Mackenzie, 2018).

## Importance of Reliability Revisited

Where is reliability in the cycle of instrument construction? We distinguish two uses of reliability that together cover the vast majority of applications well. First, reliability is estimated for test scores and other variables used in research directed at making general statements about human behavior. In *research*, instruments on which test scores are collected already exist, for example, intelligence tests and personality inventories, and variables can be dependent variables defined by a small number of items used in experiments. When the population in which the research is done is different from the population for which the test was developed, one needs to re-estimate reliability. For newly constructed variables, estimating reliability is an obvious requirement. Standards for minimum reliability are quite vague and can vary greatly among different authors. The provocative question of course is why reliability is important at all in research when one is rather interested in testing hypotheses about general human behavior. Does reliability relate to the power of statistical tests? If so, does a Student *t*-test require a different minimum reliability than an *F*-test? How does reduction in random error by requiring higher reliability relate to increasing sample size in statistical testing? How does reliability relate to testing goodness of fit of multivariate models to the data? Central to these and other questions is the question how reliability affects the power of the statistical tests used. Some but not much work on the relation between reliability and statistical power has been done (e.g., Nicewander & Price, 1983; Williams et al., 1995; for more recent work, see Ellis, 2013a, b). Clearly, this is a rich area for future reliability research.

Second, tests and questionnaires are used in the selection and placement of *individuals* in education (pass/fail, feedback, remedial teaching, change, admission/rejection), the job market (selection/rejection, assignment to tasks), and psychological and medical therapy (yes/no, therapy type, duration, change). The extent to which psychological and educational measurement instruments are used is impressive. Few people in Western societies have never been tested. Whereas requiring highly reliable instruments for high-stakes decisions seems self-evident, it is equally important to realize that reliability is a group characteristic, whereas decisions about individuals require reliability at the individual level. A test score with reliability .9 may not provide reliability for all individuals. Psychologists have routinely used the standard error of measurement $\sigma _{E}$ for estimating confidence intervals for people’s true scores, but depending on reliability and group test-score standard deviation [$\sigma _{E}=\sigma _{X}\sqrt{(1-\rho _{XX^{'}})}$], these confidence intervals are also group characteristics and hence have equal width for everyone. Mellenbergh (1996) called attention to the difference between the group characteristic of reliability and the scale-dependent precision of individual scores as estimates of true scores or latent variable scores (also, see Lek & Van de Schoot, 2018), which he denoted measurement precision. When precision of measurement of individual test performance is at stake, measurement precision rather than reliability is of focal interest. Future work on reliability could therefore shift in the direction of reliability’s relevance for measurement precision when assessment of individuals is of interest. IRT, which in many ways is a refining of CTT (Lord, 1980, chap. 3), estimates a scale-dependent standard error equal to the inverse of Fisher’s information function and thus is able to show for which scale values the test is relatively precise. This property is at the basis of applications such as adaptive testing using item banks.
